# Genetically Modified Animal-Derived Products: From Regulations to Applications

**DOI:** 10.3390/ani15111570

**Published:** 2025-05-27

**Authors:** Carlos Fajardo, Marta Macedo, Tonka Buha, Marcos De Donato, Benjamin Costas, Juan Miguel Mancera

**Affiliations:** 1 Department of Biology, Faculty of Marine and Environmental Sciences, Instituto Universitario de Investigación Marina (INMAR), Campus de Excelencia Internacional del Mar (CEI·MAR), University of Cadiz (UCA), 11510 Puerto Real, Spain; juanmiguel.mancera@uca.es; 2 Interdisciplinary Centre of Marine and Environmental Research, University of Porto (CIIMAR), 4450-208 Matosinhos, Portugal; up202202115@edu.icbas.up.pt (M.M.); tbuha@ciimar.up.pt (T.B.); bcostas@ciimar.up.pt (B.C.); 3 Institute of Biomedical Sciences Abel Salazar (ICBAS), University of Porto (UP), 4050-313 Porto, Portugal; 4 SPAROS Lda., Área Empresarial de Marim, Lote C, 8700-221 Olhão, Portugal; 5 Center for Aquaculture Technologies (CAT), San Diego, CA 92121, USA; mdedonato@aquatechcenter.com; 6 Escuela de Medicina y Ciencias de la Salud, Tecnológico de Monterrey, Querétaro 76130, Mexico

**Keywords:** CRISPR, FDA, genetic engineering, gene-editing, GMO, intentional genomic alterations

## Abstract

Biotechnological advances applied to the generation of genetically modified (GM) animals have shown the potential to develop innovative solutions for different challenges in key areas such as agriculture and human medicine. Despite its enormous potential, the deployment of genetic modification in animals, and its subsequent commercialization, does not meet the same public acceptance as GM plant-derived products, which are currently widely adopted around the world. In this review, we highlight the main examples of GM and gene-edited animal-derived products already approved by the FDA and discuss the regulatory context inherent to such processes, including the risk-based assessment analysis based on a case-by-case evaluation. Moreover, cases of GM animals already approved by other jurisdictions around the world are also discussed.

## Simple Summary

Currently, the U.S. Food and Drug Administration (FDA) has approved different genetically modified animals for various purposes, including protein production for direct human consumption, use as bioreactors for the generation of bioactive compounds, as models for human disease research, for improving animal welfare, pest control, and even xenotransplantation. These examples represent the immense potential of this approach for the generation of innovative products with a wide range of practical applications. Given such a perspective, this review is focused on exposing an in-depth description of the current legislative framework applied by the FDA for the regulation of these types of products.

## 1. Introduction

The process of alterations of plant and animal species for human benefit has been one of the main principles of agronomic history [1]. Centuries of domestication facilitated by the gradual selection of polygenic variations lead to new generations of organisms characterized by enhanced desired traits, such as improved yield or growth rate [2]. On the other hand, transgenesis brings a new and comparatively more efficient approach for the introduction of targeted genetic variation that can also produce profound alterations in the phenotype [3]. More recently, new gene editing methods are promising to enhance and accelerate the development of economically valuable traits in different species [4].

The enhancement of agricultural species by the modification of specific features produced by genetic modification techniques became feasible in the late 20th century, when a set of molecular biology techniques allowed the development of important biotechnological advances framed into the new paradigm of synthetic biology. However, despite its wide use for the investigation of different organisms, these gene transfer and manipulation technologies have yet to be completely adopted and integrated into the current breeding programs applied to commercial animal species [5].

Through the application of precise techniques for the modification of the genome, efficiently and affordably, new crops and livestock are entering into the regulatory schemes of different countries around the world for its commercialization [6]. The products derived from the agriculture biotechnology sector, primarily referring to plant products, have quickly become one of the topmost used agriculture products worldwide [6]. For instance, during the last 30 years, the production of genetically modified organisms (GMOs) of vegetal origin has been multiplied by 100 [7,8]. In specific, soy, corn, cotton, and canola, are the leading GM crops worldwide [9,10]. For example, transgenic cotton represents around 80% of the total cotton cultured around the world [9]. The evolution of this sector has been fast as the use of GMOs for agricultural purposes was first introduced in 1996 [11], and currently, 26 countries harvest nearly 190 million hectares of biotechnological crops around the world [9,10].

Since the generation of the first GM livestock during the middle 1980s [12]; various transgenic animals have been showcasing enhancements in specific traits such as resistance to diseases [13,14,15], improved meat [16,17], enhanced wool production [18], milk composition profile [19,20], growth rate [3,21], and higher neonatal survival [22,23,24], among other cases [11,25]. However, compared to the genetic modification of domesticated plants that have been rapidly extended worldwide, the genetic engineering of agricultural animals has not followed the same pattern [26]. Such a gap in the adoption of genetic engineering in agricultural animals in comparison to plants is the result of a complex interplay of biological limitations, heightened welfare and ethical considerations, more complex and stricter regulations, limited market demand due to public perception, and underlying cultural and social behaviors [27,28]. Thus, in the case of GM animals, just a handful of examples can be mentioned.

**Figure 1:**
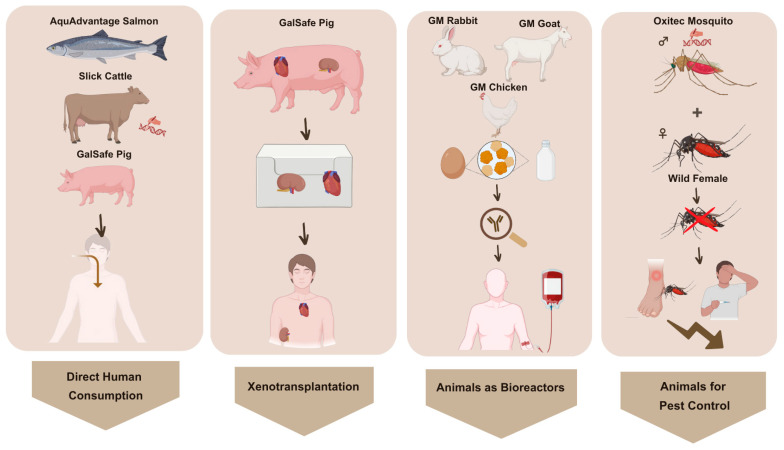
Main examples of currently FDA-listed GM animals and their applications.

In the Western hemisphere, leading bodies for GMO assessment are the FDA and the European Food Safety Authority (EFSA). Currently, it is possible to define different subcategories of GM animals already approved by the FDA: (a) those created for human consumption; (b) used as medical bioreactors (e.g., production of biopharmaceuticals, animal models for medical research, and xenotransplantation); and (c) used for pest control (Figure 1). Indeed, there are some commercially available pharmaceuticals generated from GM animals [29].

Given this perspective, this review is focused on presenting an in-depth description of different GM animals currently available, which have primarily been approved for commercialization by the FDA, focusing on their particularities and the regulatory framework applied in each case. Moreover, it describes the evolution of the legislation regarding GM animals, the considered criteria, and the role of the different political actors during this process.

## 2. Evolution and Status of the Legislation on GMOs

Following the establishment of the first GMO regulations in 1990, the evolution of the legislation about GMOs has been characterized by different phases. The following decade (2000s) was defined by a huge expansion in the commercialization of such products, which were almost entirely of vegetal origin (plants). In the decade of 2010, this process was marked by not only an increased public debate, but also by higher regulatory scrutiny by different governmental agencies at the national and international level [30]. From 2020, the evolution of this process has been deeply influenced by the rise of new technologies. In specific, the deployment of the clustered regularly interspaced short palindromic repeats (CRISPR) technique has accelerated important political advances in the regulatory framework [6,31].

The method for determining if a new plant, livestock, or product is considered a GMO is a process that has evolved to trigger national legislations, which activate different types of risk evaluations and managing strategies applied in each case. Such an assessment would depend on whether the product will be used for direct human consumption, for animal feed, or for cultivation, as there exists a specific regulatory framework. In each case, the associated risk must be evaluated and managed. Over the past 40 years, this work has been under the responsibility of the legislators [32]. Therefore, the security of the food/feed, and the environmental protection are the main criterion for the risk analysis.

Other relevant factors that need to be highlighted are the different political actors involved in this process. In this context, the main players are the governments shaping the regulatory framework about GMOs, followed by the lobby groups dedicated to the promotion of these products, and finally, the public opinion represented by the scientific community, environmental groups, and consumers [33].

It is important to highlight that, currently, there are different regulations that apply at the national level, which are translated in some cases not only into specific labeling requirements but also into the development and application of international standards related to the exportation and importation of such products (e.g., EU) [6].

Moreover, there is a clear distinction between the authorizations of GMO products for importation and exportation, for direct human consumption, or to produce animal feeds. Those distinctions are made because there are different risks linked to the culture, the commercialization, and the consumption, which require different regulatory approaches in each case. Furthermore, at the national level, diverse governmental agencies could intervene during the approval evaluation process [6]. For example, in the USA, according to the destiny of the final product, it might be evaluated by the Environmental Protection Agency (EPA), the Department of Agriculture (USDA), or the FDA, or simultaneously by more than one of these entities in some cases. Therefore, FDA, EPA, and USDA work together to ensure that GMOs are safe for human, plant, and animal health. Such agencies also monitor the impact of GMOs on the environment.

To have a general vision of the current legislative framework regarding GMOs, it is necessary to have in mind the classification and evaluation procedure based on the process and those based on products. In the first case, the regulations oriented on the process consider the technologies of genetic modification as novel in comparison with the conventional methods; thus, under this approach, importance is given to the process used to generate a new product. On the other hand, the regulations oriented toward products are based on the evaluation of the novel features of the product compared to the ones obtained by the conventional genetic improvement methods [34]. Both approaches have their strengths and weaknesses, and neither of them is considered superior to the other. Nevertheless, according to the preference of most scientific experts in the biotechnological area, the procedure based on products is generally considered the most rigorous [6].

### 2.1. Definition of GMO

The definition of GMO is given in the Cartagena Protocol about Biosecurity of the United Nations. In this document, a GMO is termed as living modified organism (LMO). According to this, a GMO or LMO is considered as such if it comprises two features: (1) the organism harbors a new combination of genetic material, and (2) such modification was introduced using modern biotechnology. In this context, the concept of modern biotechnology is considered as the use of in vitro nucleic acid technologies, such as recombinant DNA (rDNA) and the introduction of nucleic acids in organelles/cells, or the fusion of cells beyond their taxonomic group. This legal definition arises due to the necessity of establishing a clear distinction between the use of specific biotechnological technologies from those considered more conventional, for example, traditional mutagenesis, selection, and breeding methods [35].

Nowadays, there exists a renewed debate between the regulators related to the definition of genetic modification, considering the rise of new techniques for gene editing (mainly by CRISPR). Genome or gene editing involves the use of site-directed nucleases (SDNs) for developing very specific excisions into the target DNA [36]. Currently, there are five molecular tools that can be used for gene editing: (a) oligonucleotide-directed mutagenesis (ODM) [37]; (b) zinc finger nucleases (ZFNs) [38]; (c) mega nucleases [39]; (d) transcription activator-like effector nucleases (TALENs) [40]; and (e) CRISPR [36,41]. Therefore, nowadays, the regulators are more confident in a wide-range categorization of these technologies as SDN-1, SDN-2, and SDN-3, respectively [42].

Based on the above mentioned, a threshold has been established for the definition of GMO, as the SDN categorization refers to the following specific intended changes in the DNA. Accordingly, the SDN-1 technique implies that nucleases are directed to a precise sequence of the DNA to generate a single excision on the double-stranded structure of the DNA (DSB), or two DSBs to eliminate one segment of the target DNA. On the other hand, the SDN-2 implies the use of a short template of donor DNA for guiding the modification, giving as a result an intended mutated sequence of DNA. In the case of SDN-3, these techniques use a much more complex template of donor DNA, that is subsequently inserted into the target place, resulting in a modification that is very similar to those obtained by conventional rDNA methods [43].

Currently, many experts in this area refuse to consider gene editing as genetic modification, mainly because they argue that changes generated in the DNA by such techniques are not different from those induced using traditional breeding, or from those occurring naturally in the environment [41]. Such researchers claim that crops or livestock having equal phenotypes could be generated using either induced random mutagenesis or gene-edition methods [44].

Furthermore, the current scientific progress regarding the specificity and fidelity of nucleases [45], mainly the CRISPR-associated Caspase-9 (Cas9), and its different variants (e.g., SniperCas9, HypaCas9, HFCas9, eSpCas9, CjCas9, SaCas9, Cas12a, Cas13, CAST, Cascade) [46,47,48,49,50,51,52,53,54,55], has enabled the development of methods that even allow single nucleotide changes without generating DSBs [56,57,58]. Based on this fact, and in the possibility of deploying these new methods and techniques to generate enhanced crops and livestock that could avoid the expensive and time-consuming assessment linked to the commercialization of GMO products, a shift to the use of gene editing methods is increasingly tempting [41,59,60].

### 2.2. Intentional Genomic Alterations (IGAs)

Recently, the FDA defined IGAs in animals as those modifications executed on genomic DNA using modern molecular procedures, which might include targeted or random alterations on DNA, such as deletions, substitutions, and insertions [61]. Thus, IAGs applied to animals reflect the current definition of what has been previously referred to as GM animals [62]. The main difference between IGA and GMO is that the last one normally implies the use of foreign DNA, while the IGA does not [63]. However, the modification of animals by techniques that rely on the introduction of foreign donor DNA into the genome of the animal, while representing some added risks, also allows the generation of a wide range of modifications that could be translated into the development of more beneficial and innovative features, like for instance, disease resistance [45].

The IGA can be introduced into the animal genome using genome editing, recombinant DNA, or other technologies. Moreover, IGAs may be heritable (performed on germinal cells) or non-heritable (performed on somatic cells). In the case of heritable IGAs, the regulation applies to the modified genomic DNA in both founder animals and their offspring. The animal’s IGAs could have different uses, such as application in human health (e.g., reduced allergenicity, production of therapeutics, production of animals used as a model human disease), for enhanced well-being, animal health, and husbandry practices (e.g., heat tolerance, pathogen resistance), and for improved food quality and production (e.g., nutritional benefits, feed efficiency rate, faster growth) [61,62,64]. Recently, the FDA’s Center for Veterinary Medicine (CVM) published two documents that serve as guidance for the industry (GFI#187A and GFI#187B). The first one describes the risk-based approach applied to heritable IGAs in animals [63]; and the second draft the approval process applied to IGAs in animals [65].

### 2.3. FDA Criteria of Evaluation

Importantly, the FDA regulation criterion is not based on whether a new product is considered a GMO or not. Therefore, the FDA/CVM analysis of the prospective risk characteristics of a new product is not based only on the generation technique used (e.g., rDNA or gene editing) or type of the IGA (e.g., large deletions vs. single nucleotide alterations; deletions vs. insertions), but also considering other factors such as the intended use, IGA’s features, previous information about safe use, and animal containment degree [45]. Independent of the type of method used to generate the IGA, the FDA/CVM evaluation is based on the risk. Therefore, the risk-based approach for the evaluation process of these products balances between both regulatory responsibilities and the necessity to develop new products that could reach the market efficiently. In general, and according to the FDA/CVM criteria, IGAs in animals should be (a) safe for the animal itself; (b) safe for direct human consumption (if applies); (c) effective (i.e., according to the claims stated by the sponsor); and (d) FDA/CVM must analyze the potential environmental effects of the approval, stating if are significant or not. All are in compliance with the National Environmental Policy Act (NEPA) and based on a case-by-case evaluation process [45,62]. According to the current risk-based criteria followed by the FDA/CVM, if the risk represented by the potential impact derived from an IGA is indeed low, it means that the agencies do not expect that sponsors of these events need FDA approval to commercialize such products. Such low-risk events nowadays comprise IGAs generated in animals used as models for human disease research and might potentially also comprise IGAs in animals used for direct human consumption. Importantly, the amount and type of information needed to reach these standards could be different based on the risks of the product. For instance, if an IGA is generated in an animal of a food-generating species, but will not be used for food purposes, the data/information requirements regarding food safety will be less compared to an IGA in an animal that was created for direct human consumption [45].

Accordingly, if the risk is characterized as low, these agencies do not expect sponsors to ask for the FDA’s approval (either by an application, risk information, or notification) prior to marketing [45]. Such types of low-risk IGAs comprise those in highly isolated animals used for research, like mice and rats. In those cases, the FDA’s criteria consider that such products are currently well regulated or represent a low risk, mainly due to such animals not being expected to enter the food chain supply, or to be released into the natural environment. On the other hand, in the case of those events that might represent higher risks, they are expected to pass throughout the entire FDA’s approval process. In such cases, the FDA/CVM are also devoted to optimizing that process, to be more transparent and efficient [45].

To support this goal, the FDA/CVM established a Veterinary Innovation Program (VIP). The VIP is available for the sponsors of some innovative events, like most of the IGAs in animals. Such initiative offers multiple benefits like the availability of review groups, the possibility to stop and reinitiate deadlines, pre- and post-revision feedback, practical assistance, and senior consulting. Therefore, the main objective of the VIP is to support the generation of new products derived from GM animals by offering higher certitude in the review process and facilitating a predictable and efficient approval procedure. It is important to highlight that in the case of first-time sponsors, who are often academically based research initiatives or small start-up companies, such processes can be difficult to understand and carry out. Thus, the VIP was created to facilitate this process. As a result of this program, the FDA/CVM has around 25 new products registered in this initiative, and the number is continuing to increase [45]. Recently, the USA Presidential Executive Order (14081) was promulgated for “Advancing Biotechnology and Biomanufacturing Innovation for a Sustainable, Safe, and Secure American Bioeconomy”. By this instrument, both EPA, FDA, and USDA have developed a coordinated framework to clarify streamline and update their normative and surveillance procedures for biotechnological products [66].

Importantly, the FDA considers the modifications of the genome of food-producing animals as a “new animal drug” (NAD), and therefore, a new animal drug application (NADA) must be filed for these animals. Regarding the product review process implemented by the FDA for the approval of a NAD, it is possible to define three clearly defined stages: (i) Pre-investigational new animal drug (INAD), which is considered the first contact of the product sponsor with the agency in which information about the discovery and/or proof of concept is disclosed. At this point, the agency defines the jurisdiction and the regulatory pathway to be followed by a specific product. (ii) INAD, which is considered the product development phase characterized by pre-submission conferences and requirements needed for specific technical sections. (iii) NADA, considered the final stage of the review process necessary for approval and marketing. This phase is characterized by the post-approval reporting requirements [67]. During the first two phases, it could be involved other FDA centers (e.g., CVM). Importantly, in the case of GM animals used as bioreactors to produce pharmaceuticals, NADA approval is normally required prior to the human product approval, as the GM animal is considered part of the manufacturing process [67].

## 3. GM Animals for Direct Human Consumption

### 3.1. AquAdvantage^®^ Salmon (AAS)

**Figure 2:**
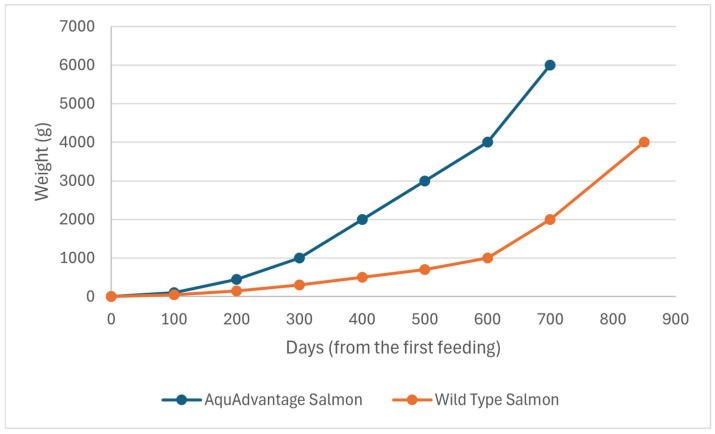
Comparative growth rate between AAS and wild type *S. salar*. As a result of the genetic modification, AAS can reach the market size in nearly half time in comparison to its wild counterparts.

This event was sponsored by AquaBounty Technologies, Inc. (Harvard, MA, USA). AAS is a transgenic strain of Atlantic salmon (*Salmo salar*), altered via the insertion of a construct designed for the expression of a supplementary gene copy of the growth hormone (GH) extracted from *Oncorhynchus tshawhytscha*, which is a different salmon species known as Chinook. As a result, AAS not only produces a higher level of GH, but also does it continuously, rather than seasonally, as in the case of the wild salmon [68]. Therefore, AAS shows quick growth during its first year of culture, which subsequently makes it reach the market size in half of the time needed for its wild counterparts [68] (Figure 2).

Furthermore, AAS needs up to 25% less feed than conventional *S. salar*, revealing a much more efficient feed-to-biomass conversion rate [69]. Such features make it economically viable to culture AAS into high-quality and fully inland recirculating aquaculture systems (RAS) [5]. Importantly, under the right conditions, AAS does not represent a threat to wild salmon populations, mainly due to the redundant physical and biological barriers developed for isolating it from its wild counterparts. Thus, the culture installations for AAS are built far from the wild salmon habitats using RAS facilities. These physical barriers of containment are further reinforced by the development of biological reproductive barriers of containment, represented by the deployment of individuals that are only sterile triploid females [70,71].

Despite the development of all these biosecurity mechanisms, the journey of AAS throughout the regulatory requirements imposed on GMOs has taken more than twenty years between the initial submission to the FDA in 1995, and its approval for direct human consumption in Canada (2016), USA (2021) [5,72], and Brazil (2021) [5]. In 2022, up to 10,000 MT of AAS were produced on the AquaBounty’s farm in Ohio (USA) [5,72]. In December 2024, due to financial liquidity problems, the company announced the closure of its only remaining operating facility, located in Bay Fortune (Prince Edward Island), including the culling of all remaining fish, and the reduction of its workforce [73]. Despite the decades of public and regulatory scrutiny this product has undergone, and the current financial situation of the sponsor’s company, it still represents the pioneer case of the development of GM animals for direct human consumption. Accordingly, it also represents a paradigmatic example of both the financial risks taken by the sponsors of these types of products and the grade of opposition generated in some sectors of public opinion.

#### 3.1.1. The Regulatory Process

According to the FDA and based on an in-depth and comprehensive study of the scientific research data, as stated by the Federal Food, Drug, and Cosmetic Act (FD&C Act), it was determined that AAS meets all the legal requirements for safety and efficacy. AAS is not only safe for direct consumption as food, but also the inserted DNA is innocuous for the GM fish, and AAS meets the sponsor’s claim regarding quicker growth [74,75]. The FDA also studied the possible environmental influence of AAS on the quality of the human environment in the USA [76], and issued the respective environmental evaluation and Findings of No Significant Impact (FONSI) [76,77].

#### 3.1.2. More than 20 Years of Regulatory Journey

The first line of AAS was created in the late 90’s (1989) [78], and in 1995, an INAD application was started in the FDA. Eight years later, in 2003, the FDA initiated the review and study for this NADA. In 2008, the FDA approved the AquaBounty’s hatchery facility in Canada and the company started the construction of an inland farm facility located in Panama. By 2009, the sponsor company has already submitted more than 25 research studies to support the FDA application. In 2010, the FDA’s findings were exposed to the Veterinary Medical Advisory Committee (VMAC), and approval was recommended. The VMAC, integrated by selected independent experts from industry, non-governmental organizations, and academia, agreed with such findings, and concluded that AAS is equivalent to the traditionally farmed *S. salar*, and is safe for direct human consumption, as well as innocuous for the natural environment [72]. In 2012, the FDA released both the draft of the environmental risk analysis for public debate, and a FONSI, stating that growing AAS in those specific locations did not pose any risk to the natural environment when GM fish are maintained under the strict conditions indicated by the FDA [70,72].

In 2015, AAS was approved for direct human consumption in the USA [74]; nonetheless, in 2016 the FDA announced an Import Alert forbidding the introduction of AAS into USA territory until labeling requisites were stated. In 2016, Health Canada approved AAS for direct human consumption, sale, and production in its territory. In 2018, the FDA approved the raising of AAS at the sponsor’s facility located in Indiana, USA [79], and a second harvest of AAS grown at the Panama facility was sold in Canada. In 2019, the FDA eliminated the Import Alert, enabling the introduction of AAS eggs into the USA; while the Environment and Climate Change Canada (ECCC) endorsed the sponsor’s farm located at Rollo Bay (Prince Edward Island) for commercial production. The first harvest of AAS in the USA and Canada occurred in 2021; and during that year, Brazil approved AAS for direct human consumption [5,72].

#### 3.1.3. Difference Between AAS and Other Fish

**Figure 3:**
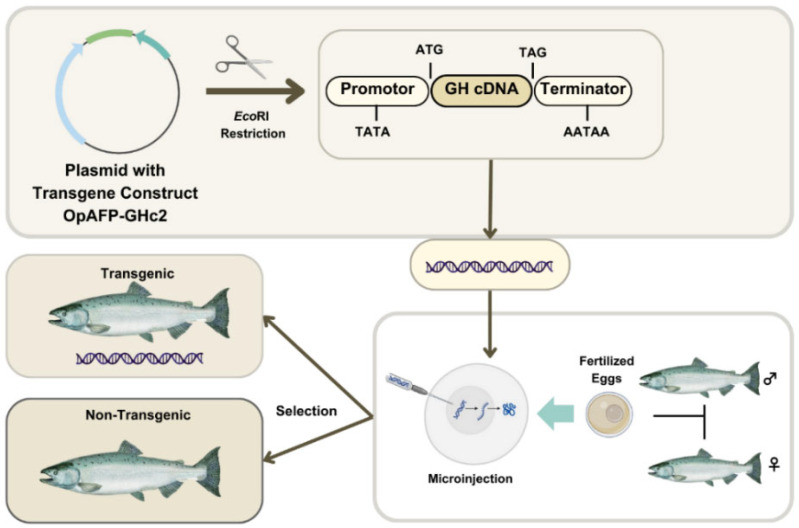
Generation process of AAS. The rDNA construct (opAFP-GHc2) is microinjected into fertilized eggs to obtain the transgenic founders. Image modified from Walton et al. [72].

**Figure 4:**
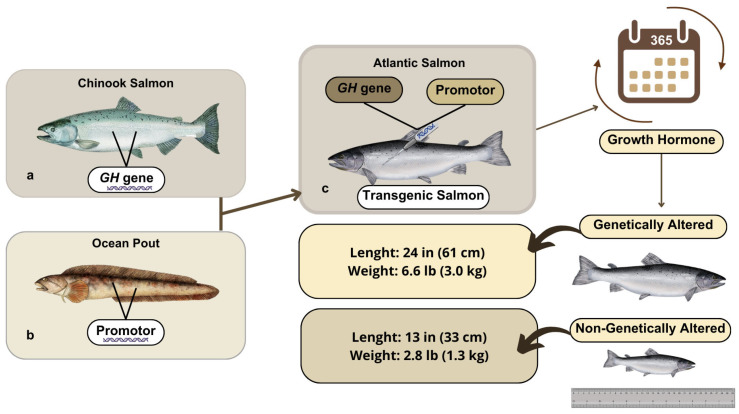
Design process of AAS. GH gene from Chinook salmon (**a**) is ligated to a promoter derived from the ocean pout (**b**). Integrated into Atlantic salmon DNA (**c**), recombinant GH gene construct controls the expression of GH during all year instead of only in summer. As a result, AAS can reach the market size (4–5 kg) in nearly half of the time (16–20 months) in comparison to their non-GM counterparts (28–32 months).

AAS has been modified to achieve a key feature searched by the aquaculture industry: a faster growth rate than its non-GM farm-raised counterparts [71]. In specific, AAS was developed through the insertion of a GH transgene (opAFP-GHc2, termed EO-1α) [80], by microinjection [81], into fertilized Atlantic salmon (*S. salar*) eggs (Figure 3). The EO-1α transgene [80] was built from the GH gene (GHc2) of a similar species, the Chinook salmon (*Oncorhynchus tshawytscha*), under the control of the ocean pout (*Macrozoarces americanus*) op5a anti-freeze protein gene promoter (opAFP), which was truncated and re-located at 5′ region of the GH gene [82] (Figure 4). The AAS genetic rDNA construct confers upon the non-GM farm-raised Atlantic salmon not only a quicker growth phenotype, but also an enhanced metabolic rate linked to a low but steady level of GH expression. As a result, the fast growth of AAS allows it to achieve the market size in nearly half of the time compared to traditional *S. salar*. Significantly, AAS grows to 100 g body weight than their non-GM counterparts within 2700° days post-hatch. Indeed, AAS reaches smolt and market size ~5 fold and ~2 fold faster compared to conventional Atlantic salmon. AAS is commercialized only as sterile all female (triploids) eyed eggs, and GM fish must be strictly contained in freshwater at inland physically isolated installations [70].

Arguably, fish have been one of the groups of species subjected to higher efforts to modify their features to improve yields; and ultimately, expand the aquaculture industry. Nowadays, more than 33 fish species have been genetically altered with a plethora of constructs engineered to modify their physiology and achieve different goals [83]. GM fish harboring transgenes have been generated for more than 25 years using several aquaculture species [26]. The most common examples of GM fish include those altered to over-express GH, resulting in a dramatically enhanced growth response in many species [84,85,86]. Moreover, GM fish’s potential to modify its phenotypes by natural selection and plasticity has also been described [26].

#### 3.1.4. AAS Food Safety Assessment

As part of the review and evaluation procedure, the FDA opened a conference, in which public opinions were collected and published as a draft with an environmental assessment that was publicly debated and reviewed. Subsequently, the agency stated that food products derived from AAS were safe for direct human consumption and comparable to non-GM *S. salar*. Regarding this point, the FDA also stated that the nutritional profile of AAS was comparable to non-GM farm-raised *S. salar* [71]. The FDA carried out a comparative analysis using three different fish groups: AAS, and conventionally farmed *S. salar* from both the sponsor and from another company. This analysis compared key hormones, such as insulin-like growth factor 1 (IGF1), estradiol, 11-ketotestosterone, testosterone, T3, and T4, finding no biologically relevant differences [71].

Currently, there are several methods for the quantification and detection of the growth hormone 1 (GH1) [87,88] and the anti-freeze protein (op5a) genes [89] present in food products. For example, in the case of GH1, the absolute and relative threshold of detection were 0.01 ng/μL and 0.01% of gDNA, respectively. These results show that RT-qPCR methods are suitable for the precise detection of AAS in food ingredients [87].

The development of very precise methods of detection and quantification takes more importance in the context of the labeling requirements needed in some jurisdictions. For instance, the labeling of GMO-derived products is obligatory in the EU with a limit of 0.9% according to the regulation instrument of the EC 1830/2003 but is not mandatory for GM plants in the USA. Nonetheless, the regulations of GM fish by the FDA were different regarding traceability and labeling, due to an amendment of the FD&C Act that required the labeling of this specific product (AAS).

At this point, it is also important to highlight the development of internationally harmonized principles for risk assessment of foods derived from rDNA animals by the Codex Alimentarius Commission (CAC) [90].

#### 3.1.5. Labeling

The USA Department of Agriculture’s Agricultural Marketing Service (USDA AMS) controls the exposure of bioengineered content on the labeling of human food containing GM fish according to the National Bioengineered Food Disclosure Standard. This normative, issued in 2018, required that human food harboring GM fish content must be labeled to indicate its bioengineered origin [71]. According to the apologists, the rise of this specific regulation was part of the last maneuvers of the anti-GMO groups, which at that point were already resigned to accept the official approval of GM fish and other GM animal-derived products. Given this scenario, anti-GMO activists desperately demanded labeling requirements. This last resort was interpreted by some market stakeholders as not only an economic extortion, but also a direct effort to vilify future food products derived from GMOs [70].

#### 3.1.6. Risk Managing Assessment of AAS

In most of the applications of GM fish, one of the most common risks managing strategies proposed is the use of confinement techniques to lower the possibility of escape of the GM fish to the natural environment, or if such exposure occurs, to lower their ability to reproduce and established with the wild populations [91,92,93]. Such risk management strategies have been proposed not only due to, in most cases, the elimination of introduced GM animal results impossible [94], but also because the degree of uncertainty linked to the estimation of the environmental impact is generally high.

##### Doble Barrier of Physical and Biological Containment

Physical Barriers

One of the main risk factors that need to be considered for calculating the likelihood of the escape of GM fish is the scale of the culture installations. Regarding this point, the physical confinement strategies can be highly effective if strict and specific construction normative and human resources management are correctly deployed (e.g., redundant barrier systems, maintenance, and biosecurity) [26]. Furthermore, land-based RAS, which are improving in sophistication and size, are considered today as the most efficient and reliable physical containment methods used to reduce the negative impact of aquaculture on the natural environment [95].

In the specific case of AAS, and according to the specific regulations of the FDA, those GM fish need to be subject to strict isolation conditions to avoid the possibility of escape into the natural environment. These GM fish, under any circumstances, cannot be grown in open ocean net pens. Instead, the FDA only approved its growth into very specific land-based installations: one in Canada, used for maintaining the breeding stocks; and another located in Indiana (USA), where the fish intended to sell on the markets would be grown using the eggs from the Canadian installations [71]. The FDA has carefully reviewed the Canadian and USA installations of the sponsor company. Based on the inspections of these installations, the FDA stated that not only that the likelihood of GM fish escapes is very low, but also that the strategies deployed to monitor physical isolation are robust. Additionally, the Canadian authorities also inspected regularly the sponsor facilities in their jurisdiction [71].

Moreover, at the grown-out facility in Panama, built more than 100 km from the ocean, all the AAS individuals (all females, sterile, and triploids) were maintained in isolation in highly bio-secure grown installations, having in total twenty-one individual confinement barriers, with at least eleven screens deployed in series. Supplementary to the physical and biological barriers, a natural thermo-lethal barrier of warm water downstream of those installations, would prevent any potential escaped live AAS could reaching the Pacific Ocean [70]. At this point is important to highlight that *S. salar* (AAS or conventional), as a cold-water fish, could not survive in waters with a temperature higher than 25–28 °C [96,97], being their optimal growth temperature around 16–17 °C [98], and the lower and upper-temperature limits between 6 and 22.5 °C [99].

To our knowledge, currently, there is no record of the introduction of GM fish into the natural environment. Nonetheless, considering the hypothetical case of a breach in the confinement, or even in the case of an intended introduction (e.g., framed in the context of invasive species control species) [92,100], both reproductive and survival capacities of the GM fish would play a fundamental role over the magnitude and duration of any resulting impact. Thus, the development of an additional defense line, represented by supplementary biological isolation barriers, is highly recommended [26].

Biological Barriers

The two main approaches to prevent or limit the effects of potential escapes, are firstly the localization of the culture installations in zones where the scaped GM animals cannot survive in the surrounding environment and, secondly, controlling the reproductive capability of the GM animals, either by using mono-sex (single-sex) animals maintained in isolation or by the direct sterilization of the GM animals [93]. The sterility status can be produced in different aquatic organisms through physical procedures, such as temperature and pressure [101]. Indeed, in the specific case of the AAS cultured for direct human consumption in the USA, those animals will be all-female, and reproductively sterile (triploids) [71].

Nowadays, the simpler sterilization method applied is the induction of triploidy [102], which, in the case of several fish species, avoids the development of functional and mature gametes, both in female and male animals. Whereas in sterilization, as in the case of single-sex containment approaches, the impact would be limited to the generation linked to the escaped animals [26].

Sterility induced by triploidy, although not infallible, is generally considered the most robust biological barrier available to prevent or reduce the likelihood of interbreeding between farmed and wild populations. Furthermore, the usefulness of any containment method would depend on its efficacy. For instance, in the case of triploid fish, farm-scale studies have revealed that very high levels of sterilization (98%) can be routinely produced, reaching even 99.8% when both induction and monitoring strategies are deployed [103]. On the other hand, in those cases in which only a few escapees are expected, or other kinds of disability are anticipated, for instance, an impaired survival, or the lack of suitable mates in the natural environment, an error rate level that produces just 0.2% diploids would generate enough containment efficacy [26].

#### 3.1.7. Evaluation of Environmental Effects

In comparison to the risk analysis of GM plants, which are generally created from long-domesticated species with a lower capacity to establish in the natural environment, GM fish are normally generated from strains that are very close to their wild counterparts, and therefore, have an ideal capacity for interbreed, both with themselves and/or with wild individuals. Based on this fact, GM fish have a tremendous scope for establishment in nature if they can escape from different barriers of containment, both abiotic and biotic. However, in most cases, GH transgenic fish are expected to have an equivalent or reduced fitness status in comparison to their wild-type counterparts [104], mainly because their highly modified phenotypes have not evolved due to a natural selection process [26].

In the specific case of AAS, and according to the guidelines of the NEPA, the FDA assessed that the AAS application approval would not produce any significant negative influence over the human environment in the USA. Based on the diverse biological and physical barriers of isolation proposed and deployed by the sponsor company, the agency concluded that the approval of the AAS application would not produce any significant effect on the natural environment in the USA. This conclusion was supported by the very low probability that AAS could escape, survive in the natural environment, and interbreed with wild *S. salar*. Such a statement was established mainly due to the different and redundant isolation strategies described in the application. Therefore, based on the agency’s findings regarding environmental assessments, the FDA issued a FONSI [71]. Moreover, the regulatory procedure included an environmental assessment of both the AAS egg production facilities in Canada, and the grow-out site located in Panama, which were also reviewed and approved by the FDA. In those facilities, there were included physical, biological, and environmental measures of containment [70]. Currently, it is available a document that states the environmental risk analysis criteria applied in the case of GM fish intended to be commercialized on the EU markets [105,106].

#### 3.1.8. Consumer Acceptance and Public Perception of AAS

Being the first case of a GMO animal approved for direct human consumption [5,71], AAS has gained a lot of attention, both from apologists and detractors. The opposition came mainly from the anti-biotech, and anti-GMO organizations, and from the organic food industry, whereas, according to the apologists, AAS is considered the most efficient and cost-effective food production option offered by the GMO industry (including plants and animals) [70].

The potential applications of GM fish have created significant public and scientific concerns related to not only the economic benefits, but also the possible risks in the areas of food and environmental safeness, as a result of their introduction and impacts on both human food supply and nature [26]. Nonetheless, despite early controversies, many biotechnology advances have been widely supported in fields such as medicine, and even in some specific aspects of the food production chain, for instance, in the case of beverage fermentation, and cheese-making processes. However, the consumer perception and acceptance of other kinds of GM food products, such as corn and soybean, remain polarized, mainly due to the prolonged campaigns of both anti-GMO organizations, and the organic food industry, which, according to some authors, are intended to vilify these products for their own economic profit [107].

Currently, the general scientific opinion, supported by more than 40 years of research and experience, points out that GM food/feed, widely used nowadays, is as safer for agricultural animals and humans as their conventional counterparts. However, the consumer acceptance and perception of food products harboring GM components remain mixed [108]. For instance, recently, an in-depth study focused on the consumer perception of GM food carried out in China showed a higher acceptance rate of this type of product when the consumers were more detailed informed about their potential advantages [109]. Thus, by the statement of such benefits, the developers can influence the consumer perception to obtain a higher rate of acceptance by a public that is increasingly cognizant and concerned regarding issues like environmental and biodiversity protection [5]. In another example, the data obtained by a recent study indicate that both Norwegian and USA consumers not only acknowledge the significance of using bioengineering to enhance both farm animals and plants but also show their willingness to purchase AAS [110].

### 3.2. Slick Cattle

Recently, an IGA deployed to truncate the prolactin receptor gene (*PRLR*) [111] in a lineage of cattle (*Bos taurus*), resulting in a short slick pelage in the gene-edited animals generated (PRLR-Slick Cattle). This IGA is a heritable modification [63] that was developed using gene-editing (CRISPR) in two founder beef calves [112]. Importantly, in this case on specific, it was considered that such IGA is equivalent to a conventional mutation occurring in normal cattle, which is considered an adaptation to being farmed in a subtropical or tropical climate. This slick mutation produces a phenotype characterized by a short and slick pelage, such animals being more resistant to hot weather conditions [113]. This event was sponsored by Acceligen, Inc. (a Recombinetics company).

Cattle with slick mutations have been reported to be more resilient to warm weather conditions [114,115,116,117,118,119]. Therefore, the development of this trait acquires more importance given the perspective of the climate change scenario [120]. Indeed, the study of the information provided by the sponsor’s company, as well as other data considered by the FDA, demonstrated that such gene-edited cattle are equivalent to their conventional counterparts presenting a natural mutation that occurs in normal cattle that has a long previous record of safety use for direct human consumption. Thus, such natural mutations produce the identical phenotype observed in the IGA cattle [113].

However, in this case, both the sponsor’s and FDA’s assessments of the genomic information revealed evidence of not expected mutations in the founder animal. Nevertheless, based on the type of such unintended mutations, it is expected that it does not cause any changes in the protein expression [121]. It is important to highlight that developers need to recognize potential side effects and evaluate possible associated issues. The rise of side effects in a gene-edited animal does not necessarily mean, a priori, that the IGA is not safe, or that the product derived from it should not be approved to enter the market. In such cases, the unintended effect needs to be well characterized to state if represents, indeed, deleterious consequences. Important improvements include the development of standardized assessment methods (e.g., for unintended effects identification). In this regard, it highlights the initiative of both the National Institute of Standards and Technology, and the Genome Editing Consortium, which are the key players in the characterization of IGA-derived products developed by gene-editing methods [45].

#### Risk Assessment of Slick Cattle

Based on the analysis of both animal health information and molecular description, the FDA stated that PRLR-Slick Cattle do not represent any safety issues for themselves or for consumers [113]. However, some researchers have expressed concern about potential welfare issues; mostly due to the pleiotropic nature of the prolactin gene, which could affect the hepatic function of these gene-edited animals [64].

Additionally, the FDA also stated that PRLR-Slick Cattle do not represent any environmental hazard, as the IAG-developed trait is a well-established trait in many domestic cattle normally raised in the USA [64,113]. Indeed, the environmental risk identified was very low, mainly due to the low probability of escape. In this case, it would be easier to rapidly recover any escaped gene-edited animal, and no wild cattle populations are present in the environment of the USA [113].

Based on the abovementioned, the FDA concluded that gene-edited cattle, and related products, such embryos, semen, offspring, and derived food products, represent a low risk to consumers, animals, the food supply chain, and the natural environment. In this case, the FDA’s conclusions are restricted to the commercialized products (embryos, semen, live gene-edited cattle, and meat) derived from the only two existing gene-edited cattle harboring the IGA for which the FDA has evaluated information and their offspring. Moreover, the FDA will regulate the farms/installations engaged in ordinary agricultural practices for these gene-edited cattle, such as assisted reproduction methods or food production, in the same way as the farms/installations dedicated to these activities for conventional cattle [113].

It is important to highlight that in this specific case, in contrast to the more than 20 years of regulatory process of both AquAdvantage^®^ Salmon and GalSafe^®^ Pig, PRLR-Slick Cattle were approved by the FDA much more quickly. In the latter case, the FDA argued that as the gene-edition introduced was identical to an already existing trait that can be found in conventional cattle, it was not necessary to carry out further assessments. Because of this, the agency also stated that it was not necessary to include any additional labeling requirements over meat, or the offspring derived from such gene-edited animals. However, this decision would not be interpreted as the FDA is now exempting all gene-edited animals from the longer approval process, rather such a decision should be considered by a case-by-case evaluation process. It is expected that in 2024, this product will be available in supermarkets in the USA [64,113,122].

## 4. GM Animals for Multiple Purposes (Direct Human Consumption and Xenotransplantation)

### 4.1. GalSafe^®^ Pig (GSP)

One very interesting approach is the generation and use of GM animals with the objective of enhancing the suitability of animal organs used for xenotransplantation [123]. Recently, the FDA approved an IGA in a strain of farmed pigs (*Sus scrofa domesticus*) [124] termed GalSafe^®^ Pig (GSP). This event was sponsored by Revivicor Inc. (Blacksburg, VA, USA, formerly PPL Therapeutics), now a subsidiary of the United Therapeutics Corporation.

**Figure 5:**
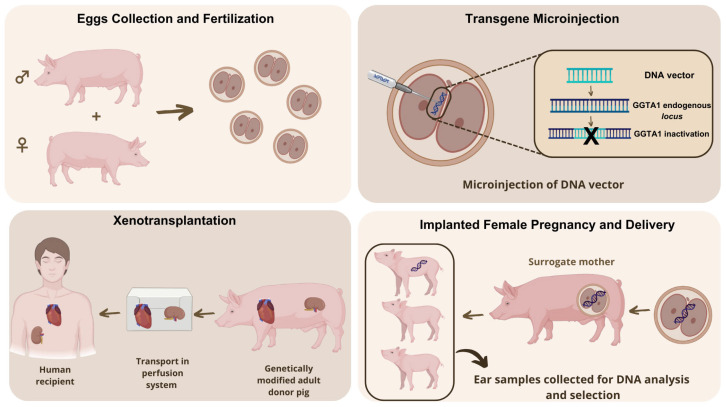
Generation process of GSPs. These animals are created by micro-injecting rDNA construct into fertilized eggs (pronuclei). The genome integration and disruption of GGTA1 gene is mediated by homologous recombination. Gestation is achieved by transferring the embryos to the oviduct of surrogate mothers. Around 10% of the live births produce transgenic animals. Inactivation of GGTA1 gene, responsible for producing alpha-gal sugar (antigen), allows the generation of food that can be consumed by people who suffer from alpha-gal syndrome (AGS). Moreover, GSP-derived organs (e.g., heart, kidney) could be used for xenotransplantation. Image modified from Peterson et al. [126].

The GSP lineage has a gene disruption that translates into the suppression of the epitope galactose alpha-1,3 galactose (alpha-gal). In this case, the IGA was accomplished through the insertion of rDNA into the genome to inactivate the gene encoding the glycoprotein galactosyltransferase alpha-1,3 (GGTA1) (Figure 5). GGTA1 encodes the protein responsible for producing alpha-gal sugar (galactose-alpha-1,3-galactose). This compound is normally present on the surface of biological structures like organs, tissues, and cells of all mammals, except in humans and some other primates. Furthermore, GGTA1 was knocked out in both alleles in homozygous GSPs, which results in the elimination of alpha-gal sugar in their organs/tissues/cells. Importantly, pigs with disrupted GGTA1 pass this trait to their offspring by normal animal breeding [124], being in this case a heritable IGA [63].

Interestingly, this represents the pioneer case of a gene-edited animal approved by the FDA for direct human consumption and is currently waiting for the final approval of the agency for therapeutic uses, such as xenotransplantation [125,126].

In this case, gene edition was applied to suppress the presence of alpha-gal carbohydrates over the pig’s cells/tissues/organs, eliminating the risk of suffering severe allergic response in consumers with alpha-gal syndrome (AGS) [127]. AGS patients can develop a severe allergic response to alpha-gal sugar present in different types (e.g., beef, pork, lamb) of red meat [128]. Importantly, the product sponsor points out that during the first phases, it is intended to commercialize the meat from GSPs by direct order, rather than using retailers [127].

Moreover, GSPs may represent a potential source of porcine-based biological materials, such blood-thinning drug heparin, to generate human medical products that do not have alpha-gal sugar. Thus, by using organs and tissues derived from GSPs, it would be possible to address the issue of immune rejection associated with the process of xenotransplantation, as the presence of alpha-gal sugar is one of the causes of rejection in some patients [126].

Currently, there are tremendous limitations regarding the availability of human organs for transplantation, especially in the case of hearts and kidneys [129,130,131]; thus, this novel product could also help to alleviate this problem. For instance, around the world, around 800 million patients suffer from chronic kidney disease, and from those, around 1 million lose their lives every year due to renal complications [132,133]. Indeed, kidney failure is the 10th main death cause in the USA, leading to around USD 87 billion in medical-related costs, renal transplantation therapy is responsible for most of this burden [134]. According to the estimations of United Therapeutics Corporation, the Health Resources and Services Administration, and the United Network for Organ Sharing (UNOS), in the USA, around 600,000 patients suffer from end-stage renal disease, and nearly the same number of patients have end-stage cardiac disease. Moreover, around 105,000 people are waiting for transplants [135], while nearly 25,000 transplants of kidney [136], and 3800 heart transplants are carried out each year in the USA. Indeed, around 6000 persons die every year in the USA waiting for organ transplantation [126].

### 4.2. Environmental Risk Assessment as Food Product

As part of the regulation procedure, the FDA assessed the possible effects of GSPs on the environment of the USA, concluding that it is not higher than those from normal pigs. Indeed, the conditions for the maintenance of GSPs are much stricter than those for normal pigs. Moreover, no more animal safety issues were identified for GSPs beyond those normally expected from conventional swine commercial operations [137].

### 4.3. Food Safety Assessment of GSP

Furthermore, the FDA also stated that microbial food safety risk (represented by antibiotic-resistant bacteria strains, both in humans and pigs) is low and mitigated not only by the small number of GSP individuals integrated into the food chain supply, but also by the periodical surveillance mechanisms deployed for the detection of antibiotic resistance [137]. According to the FONSI statement carried out by the FDA, no more than one thousand GSPs/year will be generated in a specific installation located in Northern Iowa and sacrificed in another facility in South Dakota. Both installations are periodically inspected by the USDA [138].

### 4.4. Timeline of GSP Development

In 2000, the company PPL Therapeutics (named Revivicor Inc. in 2003) generated the first cloned pigs [139]. In 2001, the pioneer alpha-gal knockout pigs were developed, which were the progenitors of the GSP lineage [140]. In 2011, Revivicor was purchased by United Therapeutics Corporation with the goal of creating a supply source of manufactured organs for use in transplantation procedures. In 2020, the company received FDA approval for using GSPs as both food for direct human consumption and for producing therapeutics intended to be used in humans [124,137].

In 2021, a UKidney™ extracted from a GSP was transplanted into a recently deceased human body connected to external support [135,141]. In 2022, a UHeart™ was clinically-used establishing a record with the longest-to-date surviving patient after receiving a xenotransplantation [137,142]. In 2023, a UThymoKidney™ established another longest-to-date record for a GM pig kidney working in a human body. Moreover, a UHeart™ was clinically used in a second human recipient. In 2024, an in-depth review was published describing the current state of the art regarding xenotransplantation. The review summarizes not only the historical background of the techniques used to create GM pigs but also gives insight into the research currently conducted on the xenotransplantation of kidneys and hearts extracted from GM pigs [126].

Importantly, both UKidney™, UHeart™, and UThymoKidney™ are not approved for commercialization in any jurisdiction yet, as they are currently considered development-stage products. Indeed, GSP has not been completely evaluated yet for its use as a xenotransplantation product. For these purposes, it is necessary to obtain additional FDA approval and carry out a separate environmental analysis according to the NEPA, before such product can be commercialized and used in human medicine [127,138]. However, this is the first approved case of a GM animal-derived product created to be used as both a source of food for direct human consumption and for biomedical applications, representing an important milestone for this type of biotechnological innovation.

## 5. GM Animals as Bioreactors

Another approach implemented through animal bioengineering is molecular farming, also called “*pharming*”, which consists of the generation of biopharmaceuticals using GM animals [143]. In comparison to recombinant cell cultures, animals could be more attractive bioreactors, for several reasons, including (i) the presence of the right metabolic pathways; (ii) better reproducibility (iii) easier maintenance; and (iv) no need for expensive facilities [144]. For instance, to produce recombinant proteins, normally the milk of mammals is used. This method has several advantages, like flexible production, and makes relatively easier the process of purification. Besides milk, the seminal plasma and egg white have been used for this purpose [144]. In the case of blood, usually, this tissue is not a good option, as it cannot store higher concentrations of recombinant proteins [145].

### 5.1. Different Types of GM Animals Used as Bioreactors

Recently, the FDA approved the NADA 141-511, regarding the generation of GM rabbits engineered to produce recombinant human factor VII (rhFVII), which is used for the treatment of patients (adults and adolescents) suffering from hemophilia B or A using inhibitors of factors IX and VIII. The review process, carried out by the FDA/CVM, considered the effectiveness and safety data presented by the sponsor for supporting the NADA, and followed a multi-step, risk-based, and hierarchical process of assessment, as it is described in the Guidance for Industry 187 [146]. In this case, the specific construct of rDNA (Bc2371) was integrated at a specific *locus* on the chromosome 3p1.1–2 of a diploid linage (R69) of homozygous and hemizygous New Zealand white rabbits (*Oryctolagus cuniculus*). Such a construct induces the expression of the human gene encoding factor VII in the mammary gland of rabbits. By this approach, the zymogen rhFVII, which is present in the rabbit’s milk, can be purified and activated more easily. Moreover, this is an example of a heritable construct strategy, which means that the offspring of the altered rabbits inherit the intended trait without the necessity of further modifications. This product was sponsored by LFB USA, Inc. (Framingham, MA, USA) [146].

At this point it is necessary to highlight that in the case of these products, intended to be used and commercialized as human biological material, it required a separate approval, through a biologics license application (BLA), which is evaluated by the FDA’s Center for Biologics Evaluation and Research (CBER). Once the sponsor obtains BLA authorization (or any other approval) from the CBER, it is possible to legally distribute biological material as a treatment for human disease [146].

Similarly, the FDA approved a specific line of domestic goats (*Capra aegagrus hircus*) to produce human antithrombin (NADA 141-294), commercialized by the name ATryn^®^. In this case, GM goats harboring five copies of a rDNA construct (Bc6) inserted at GTC 155-92 *loci*, for the expression of the human gene that codifies for the antithrombin protein in the mammary gland of the GM goats [147]. This event was created and sponsored by GTC Biotherapeutics, Inc. (Framingham, MA, USA) [148]. Indeed, this was the first biological product derived from GM animals approved by the FDA [147].

Another example is the FDA-approved NADA 141-453, sponsored by Alexion Pharmaceuticals Inc., which describes the production of recombinant human lysosomal acid lipase (rhLAL) in egg whites of GM chickens. In this case, the rDNA construct generated (hLAL) was inserted, as a single copy, at a specific locus in a diploid line of domestic chickens (*Gallus gallus*). This construct induces the expression of rhLAL, which is intended to be used as a treatment for humans. It is important to highlight that the food or feed that can be derived from this type of product (intended to be used only for human medicine) is forbidden to enter the food or feed supply chain [149].

### 5.2. GM Animals Used as Models for Human Diseases

Additionally, there are some other cases related to the development of GM animals intended to be used as models for medical research applied to different types of pathologies (e.g., neurodegenerative and neuromuscular disorders, heart disease, cardiac arrhythmia, cystic fibrosis, cancer, etc.), including testing the efficacy of new drugs and devices [150]. One good example of this type of application is the FDA-approved GM animals developed by Exemplar Genetics, LLC, Recombinetics, or Sus Clinicals, mostly using miniature swine species (*Yucatan* and *Ossabaw*). Currently, there are more than 14 events commercialized by these companies in the USA.

## 6. GM Animals for Pest Control

### 6.1. Oxitec Mosquito

Another interesting case was sponsored by Oxitec, Ltd. (Abingdon, UK), regarding the generation of GM mosquitoes (*Aedes aegypti*) (OX513A) with the objective of suppressing the population of this species at the release site [151]. *A. aegypti* is known to be the vector of transmission of important human viral diseases, such as yellow fever (YFV; Flavivirus), Zika (ZIKV, Flavivirus), dengue (DENV, Flavivirus), and chikungunya (CHIKV, Alphavirus) [152], and a problem exacerbated by global warming [153].

Open field trials using these GM mosquitoes (OX513A) have been conducted in Malaysia, the Cayman Islands [154], Panama, Saba Island [155], Brazil [156], and the USA [157]. Considerations about the potential environmental risk assessment of this application are also available [158]. Moreover, there is no evidence that OX513A mosquitoes released into the environment would cause more harm than their non-GM counterparts [154].

### 6.2. Risk Assessment of Oxitec Mosquito

In this occasion and given the nature of the claims and objectives reinvindicated by the sponsor, the regulatory process review was conducted by the FDA/CVM, the Center for Disease Control and Prevention (CDC), and the EPA. Such evaluation included the environmental impact assessment for conducting a field trial in Florida (Key Haven) [157], and a FONSI statement [159], concluding that the intended trial will not have significant impacts on the environment. Importantly, this conclusion of the USA agencies does not mean that GM mosquitoes are approved for commercialization. Instead, approval only allows its deployment in a very specific area, under the responsibility of the sponsor company, and with strict supervision by local authorities [157].

The event OX513 was created by the single integration of a rDNA construct controlling the expression induction of an insect-optimized tetracycline repressible trans-activator protein (tTAV), to generate conditional lethality and lower survival rate in the resulting offspring [157,160]. Moreover, such a rDNA construct also leads to the expression of RFP, to facilitate the detection of these GM-mosquitoes [157]. In this case, this “self-limiting” trait approach of conditional lethality prevents offspring inheriting the rDNA construct OX513 from surviving to sexual functional adult in tetracycline absence [157]. Basically, the main idea behind this concept is that sterile males compete with conventional males for female mosquitoes. Then, if females mate with sterile males, the offspring would be lower, and the population of mosquitoes would be reduced over the next generations. Using this approach, it was possible to reduce insect populations to very low levels [161]. This and other sterile insect techniques (SITs) have been successfully applied for more than 70 years, as tools for the control of different plant and animal pests [161,162].

### 6.3. Specific Regulation for GM Mosquitoes Related Products

Recently, the FDA released a document for the regulation of mosquito-related products [163], that defines which products would be regulated by the FDA, and EPA, respectively. According to this criterium, the EPA would regulate specific mosquito-related products using the Federal Insecticide, Fungicide, and Rodenticide Act (FIFRA), when the sponsor’s claims are related to controlling the levels of mosquito populations; whereas the FDA would regulate such products using the FD&C Act, when the sponsor presents another claims, like for instance, prevention, treatment, or cure for disease [163].

## 7. Cases in Other Jurisdictions

### 7.1. Japan

In addition to the FDA-approved events already mentioned, there are other examples of gene-edited animals approved for commercialization in other jurisdictions. Such is the case of Japan, where two species of CRISPR-edited fish, *Pagrus major* (red sea bream) and *Takifugu rubripes* (tiger puffer), have been approved for direct human consumption. In this case, the fish have been modified to grow faster than their non-modified counterparts. Both fish were developed by the Regional Fish Institute, Ltd. in collaboration with the Kyoto, Kinki, and Kindai Universities, the Ministry of Health, Labor and Welfare, and the Ministry of Agriculture, Forestry and Fisheries. Indeed, such gene-edited red sea bream was the world’s first gene-edited animal for direct human consumption developed through a national initiative instead of a private company [164,165].

The gene-edited tiger puffer has the leptin receptor gene knockout, resulting in fish’s increased appetite and fast weight gain. Gene-edited pufferfish grow faster and are 1.9 times heavier than the non-edited puffers during the same farming time. This will allow production in shorter periods than non-modified tiger puffers, which require more than 24 months to grow. In the other case, the gene-edited red sea bream had the gene myostatin knockout, which suppresses muscle growth. This causes the gene-edited fish to grow larger than their conventional counterparts using the same amount of feed. The red sea bream lacking the myostatin gene has an edible part 1.2–1.6 times higher than their non-edited counterparts, with an improved feed use efficiency of approximately 14%. Therefore, both traits were developed to lower farm production costs [164,165].

According to the legal framework implemented in countries like Japan, Argentina, Australia, and New Zealand (which have transposed the definition of LMO to their own national legislations), it considers certain gene-edited animals, with mutations that could have also been generated through conventional/natural means, as non-LMO and hence exempt from GMO legislation. Yet, applicants still need to prove that their gene-edited products comply with these criteria before a national committee will designate them as exempt [6].

### 7.2. Argentina

Another interesting case comes from Argentina, where the company Kheiron Biotech has developed the first gene-edited horses (*Equus ferus caballus*). In this case, CRISPR-edited horses were modified in such a way that their muscle fibers present a faster contraction, giving them greater reactivity and speed. These gene-edited horses comply with the current Argentinian legislation as they are not considered GMO or genetic doping [166]. Since 2011, Argentinian authorities issued that gene-edited products without base pair inserts would not be considered GMO and, therefore; fall outside of the current Argentinian regulatory framework for this type of product. Conversely, products with large DNA insertions should be regulated according to the GMO framework. Furthermore, gene-edited products with only a few base pair inserts would be regulated on a case-by-case approach if their development implies the introduction of coding sequences [167].

### 7.3. EU

Another example is represented by GM frog (*Xenopus laevis*) and GM fish (*Oryzias latipes*) used as biosensors to detect endocrine-disrupting chemicals (EDCs) in wastewater [168,169]. These GM frogs and fish were modified by introducing the GFP jellyfish gene that causes them to glow when these GM animals are exposed to EDC. Therefore, the more polluted the water sample, the more they glow. This represents a practical and commercially viable tool for evaluating water safety [170]. These events were developed and commercialized by the French company Laboratoire WatchFrog.

The EU has a very cautious approach to these types of products, especially those created for direct human consumption (GM food). In 2012, the EFSA GMO panel stated that despite some gene-edited products being different from those obtained by conventional trans-genesis or cis-genesis used for the insertion of DNA into specific regions of the genome, GM food must be assessed under the European Commission (EC) regulation (Regulation EC No 1829/2003; and Directive 2001/18/EC) [167,171]. More recently, in 2018, the European Court of Justice (ECJ) stated that organisms modified through site-directed mutagenesis, such as CRISPR/Cas9, must be deemed as GMOs. According to this decision, the size or type of alteration to the genetic material is irrelevant. Consequently, if there is mutagenesis, directed or random, small or big, the organism is legally considered a GMO under the EU jurisdiction [6]. Importantly, the EU considers mutagenesis (including gene-editing) as a genetic modification; however, exempts organisms obtained with random chemical/radiation mutagenesis from the scope of GMO regulation, as there is already a historic record of safe use of these techniques in plant breeding [6].

Importantly, EFSA does not regulate but assesses risk by providing advice to the EC on products notified for marketing through expert opinions. Additionally, it is important to highlight the extensive elaboration of guidelines for the safety assessment of GM animals by the EFSA GMO Panel [90,106,172], recently updated with opinions on gene editing of animals and synthetic biology [173,174,175]. Notably, animal health and welfare also receive attention in EFSA’s guidelines [176].

### 7.4. China

Other cases worth mentioning come from China, where researchers from the Huazhong Agricultural University and the Chinese Academy of Sciences were able to use CRISPR to generate the first spineless fish by knocking out the main genes responsible for the bone growth in species such as *Carassius carassius* (pond crucian), *Ctenopharyngodon idella* (grass carp), and *Abramis brama* (bream), respectively. Further tests showed that both fatty acid and amino acid content in this gene-edited fish meat were not significantly different from their conventional counterparts and the meat quality remains equivalent. The research will continue until the third generation of CRISPR-edited spineless fish to determine the gene trait stability [177]. According to the “Regulations on Administration of Agricultural GMOs Safety” issued by China, all gene-edited products should be considered as GMOs. However, despite the Chinese authorities strictly regulating conventional GMO products, their attitude towards gene-edited-derived products is still unclear [171].

Nevertheless, considering that China, from 2014 to 2017, represented 42% of the CRISPR/Cas articles related to the agrifood sector (more than double that USA), and 69% of patent applications for CRISPR/Cas in this sector (USA was second with 19%) [178,179,180], it would be expected that the attitude of the Chinese authorities towards agrifood gene-edited products would be more permissive in the near future, at least in the case of gene-edited crops [6]. Some authors have speculated that China could follow the USA model of assessment for gene-edited products [178], whereas others have suggested the Japanese approach as the more convenient [181].

## 8. Concluding Remarks

The current biotechnological advances, such as genome-editing techniques (mainly CRISPR), have shown their enormous potential for the generation of innovative products that tackle a plethora of public concern issues, such as sustainable food production, disease transmission, and generation of human therapeutics (e.g., recombinant proteins for disease treatment, and organs/tissues for xenotransplantation), among others. Accordingly, the FDA acknowledges the potential of these novel approaches and plays a fundamental role in supporting the development and introduction of effective and safe GM animal-derived products into the market.

It is important to highlight that, for instance, both AquAdvantage^®^ Salmon and GalSafe^®^ Pig needed around 20 years between the initial generation and the final approval by the FDA. Such a lag of time could be seen as an important drawback for the sponsors of these types of products as transferring these applications from the laboratory to the market can be a difficult and time-consuming process. For instance, some initiatives fall into regulatory or technical obstacles that restrict their progress or just run out of financing. Moreover, GM animals/plants might be approved for commercialization, but consumer skepticism impulses by decades of activist alarmism destroy the product’s demand [182]. Based on this, currently, the FDA/CVM is engaged with the sponsors so they can successfully achieve product approval in the minimum time necessary [45].

Considering this scenario, these achievements could be considered as part of a positive trend that inflexes healthy competition into the biotechnological sector, which is nowadays ruled by a handful of enormous companies. Regulatory restrictions, activist groups, and misinformed consumers still represent important obstacles to these biotechnological advances; however, the current support for gene-editing from regulatory agencies around the world has motivated I+D+i investment, allowing new firms to rise and create new products [182].

Despite that different key political actors involved in this process (regulatory entities, sponsors, and consumers) could have different perspectives, in the end, the main goal remains the same: the generation of innovative products that can enhance food production, human/animal health, and animal well-being. Therefore, the development of an appropriate research-based and risk-based regulatory framework is fundamental for pursuing this objective [45].

## Data Availability

No new data were created or analyzed in this study.

## Abbreviations

The following abbreviations are used in this manuscript:

AAS	AquAdvantage^®^ Salmon
AGS	Alpha-Gal Syndrome
BLA	Biologics License Application
CAC	Codex Alimentarius Commission
Cas9	Caspase 9
CBER	Center for Biologics Evaluation and Research
CDC	Center for Disease Control and Prevention
CRISPR	Clustered Regularly Interspaced Short Palindromic Repeats
CVM	Center for Veterinary Medicine
DSB	Double Strand Break
EC	European Commission
ECCC	Environment and Climate Change Canada
ECJ	European Court of Justice
EFSA	European Food Safety Authority
EPA	Environmental Protection Agency
FD&C Act	Federal Food, Drug, and Cosmetic Act
FDA	Food and Drug Administration
FIFRA	Federal Insecticide, Fungicide, and Rodenticide Act
FONSI	Findings of No Significant Impact
GFP	Green Fluorescent Protein
GGTA1	Glycoprotein Galactosyl-Transferase Alpha-1,3
GH	Growth Hormone
GM	Genetically Modified
GMO	Genetically Modified Organism
GSP	GalSafe^®^ Pig
IGAs	Intentional Genomic Alterations
IGF1	Insulin-Like Growth Factor 1
INAD	Investigational New Animal Drug Application
LMO	Living Modified Organism
NAD	New Animal Drug
NADA	New Animal Drug Application
NEPA	National Environmental Policy Act
ODM	Oligonucleotide Directed Mutagenesis
op5a	Anti-Freeze Protein
opAFP	Anti-Freeze Protein Gene Promoter
PRLR	Prolactin Receptor Gene
RAS	Recirculating Aquaculture Systems
rDNA	Recombinant DNA
RFP	Red Fluorescent Protein
rhFVII	Recombinant Human Factor VII
rhLAL	Recombinant Human Lysosomal Acid Lipase
SDN	Site-Directed Nucleases
SITs	Sterile Insect Techniques
TALENs	Transcription Activator-Like Effector Nucleases
tTAV	Tetracycline Repressible Trans-Activator Protein
UNOS	United Network for Organ Sharing
USDA AMS	U.S. Department of Agriculture’s Agricultural Marketing Service
USDA	U.S. Department of Agriculture
VMAC	Veterinary Medical Advisory Committee
ZFNs	Zinc Finger Nucleases
